# Vertical Profiles of Abundance and Potential Activity of Methane-Oxidizing Bacteria in Sediment of Lake Biwa, Japan

**DOI:** 10.1264/jsme2.ME11285

**Published:** 2011-12-27

**Authors:** Masazumi Tsutsumi, Hisaya Kojima, Manabu Fukui

**Affiliations:** 1 The Institute of Low Temperature Science, Hokkaido University, Kita-19, Nishi-8, Kita-ku, Sapporo, Hokkaido 060–0819, Japan

**Keywords:** methane-oxidizing bacteria, lake sediment, methane oxidation

## Abstract

Vertical profiles of the abundance, community composition, and potential activity of methane-oxidizing bacteria (MOB) were investigated in the sediment of Lake Biwa. Sediment samples were obtained from two sites at different water depths. The abundance of MOB was assessed as the copy number of the *pmoA* gene (encoding the alpha subunit of particulate methane monooxygenase), measured with quantitative real-time PCR. Abundance of the *pmoA* gene peaked in the 5–8 cm layer of the sediment from both sites. MOB community composition was investigated by denaturing gradient gel electrophoresis (DGGE) analysis of *pmoA* and 16S rRNA genes. The band patterns observed in DGGE did not significantly differ with sediment depths or sampling sites. Sequence analysis of the DGGE bands indicated the dominance of the genus *Methylobacter*. Potential activity, which was measured in the presence of sufficient amounts of methane and oxygen, decreased linearly from the sediment surface to deeper layers. These results suggest that the *pmoA* gene copy number cannot be regarded as an indicator of aerobic MOB that retain potential activity in sediments.

Methane is one of the most important greenhouse gases. Global emission of methane from lakes to the atmosphere has been estimated to be 6–16% of total natural methane emissions (1). In anoxic sediment layers of freshwater lakes, a large amount of methane is produced by methanogenesis. In the case of lakes with an oxic water column that extends down to the sediment surface, most of the methane diffused to the sediment surface is consumed by methane-oxidizing bacteria (MOB) there (6, 20, 32).

Most of the known MOB are regarded as obligate aerobes, and the majority are classified into type I or type II on the basis of membrane structures, phylogenetic positions, and other aspects (8). Almost all of the known MOB possess the *pmoA* gene, which encodes the alpha subunit of particulate methane monooxygenase. This gene is specific to MOB, and *pmoA* gene-based phylogeny is almost consistent with the phylogeny based on the 16S rRNA gene (8). The *pmoA* gene has been used as a useful biomarker for qualitative and quantitative analysis of MOB communities in various environments (19).

Many previous studies reported MOB communities in lake sediments (*e.g.*, 4, 5, 24, 26, 27). Almost all of these studies focused entirely on the oxic sediment surface, given that MOB cannot grow in anoxic sediment layers below the sediment surface. As an exceptional case, vertical profiles of aerobic MOB abundance in sediment cores were reported in a previous study performed in Lake Constance (27). In that study, the *pmoA* gene copy number and methane oxidation rate were highest at 2–3 cm sediment depth, although oxygen penetrated to only 0.35 cm in the sediment core. This might be explained by the supply of an undetectable amount of oxygen to deeper layers, but further field observation and additional experiments are required to test this hypothesis. There are only a few studies on depth-related changes in the abundance and activity of MOB in lake sediment.

In the present study, vertical profiles of abundance, community composition, and potential activity of MOB were investigated in the sediment of Lake Biwa, Japan. In this lake, approximately 90% of the methane produced in the anoxic sediment is aerobically consumed at the sediment surface before the methane diffuses to the water column (20). In the sediment of this lake, the aerobic zone is restricted to the surface (13–15), but rRNA of type I MOB was detected in much deeper sediment (14).

## Materials and Methods

### Sample collection and procedures

Samples were taken from Lake Biwa, a mesotrophic monomictic freshwater lake located in central Japan. The whole water column is oxic throughout the year. Sampling was performed on 6 September 2004 by R/V *Hakken-go* at two sites (site A, 35°23.4′ N, 136°7.7′ E, *ca* 90 m water depth; site Sh, 35°14.7′ N, 136°7.5′ E, *ca* 40 m water depth), where some studies had been conducted previously (13–15). One sediment core (4.5 cm in diameter) was obtained from each site without disturbing the sediment structure, as described previously (13). The cores were transferred to the laboratory in a cooled box. In the laboratory, each core was sliced at 0–2 cm and at 3-cm intervals thereafter downwards to a depth of 14 cm. Part of each sediment sample was kept frozen at −30°C until DNA extraction. Methane concentration in each section was determined by headspace analysis (13).

### Potential activity of aerobic methane oxidation

An aliquot (0.5 mL) of each sediment sample and 2 mL distilled water were transferred to a 20 mL vial. The slurry was vortexed for 90 s while introducing ambient air with an air pump to supply a sufficient amount of oxygen. After aeration, the vials were sealed with a butyl rubber stopper. Methane concentrations in the gaseous phase were adjusted to approximately 1,000 ppmv. These vials were incubated at 15°C with shaking (approximately 200 rpm). At each time point (0, 24, 37, 45, 62, 87, and 214 h after the initiation of incubation), methane concentrations in the vial were determined using gas chromatography (GC-8A; Shimadzu, Kyoto, Japan) equipped with a flame ionization detector. As a negative control, autoclaved sediment was treated in the same manner as the experimental samples. The temporal change in methane concentration was negligible in the negative control (data not shown). Potential activity was evaluated with the assumption that the methane consumption rate is proportional to the partial pressure of methane in the gaseous phase. The rate constant was calculated as follows, on the basis of the first-order kinetics. From the assumption,

(eq. 1) $$-\text{d}n/\text{d}t=k^{\prime }P$$

where *n* is the number of methane molecules (mol), *t* is the time (hour) and *P* is the partial pressure of methane in each vial (Pa). From equation 1 and the gas equation (*PV* = *nRT*),

(eq. 2) $$-\text{d}n/\text{d}t=-\text{d}(PV/RT)\text{d}t=-(V/RT)\text{d}P/\text{d}t=k^{\prime }P$$

(eq. 3) $$-\text{d}P/\text{d}t=k^{\prime }(RT/V)P=kP$$

(eq. 4) $$P(t)=P(0){\text{e}}^{-kt}$$

where *R* is the gas constant (8.31 J K^−1^ mol^−1^), *V* is the volume of the gaseous phase in each vial (m^3^), *T* is the incubation temperature (K), *P*(0) is the initial partial pressure of methane (Pa), and *P*(*t*) is the partial pressure of methane after *t*-hour incubation (Pa). *k* is defined as

(eq. 5) $$k^{\prime }=(V/RT)k$$

**Fig. 1:**
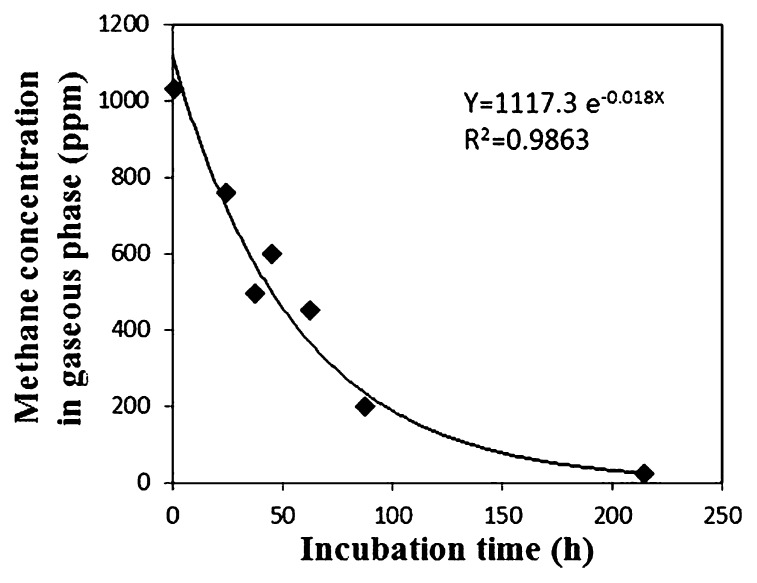
Methane consumption by sediment slurry. As a typical example, concentration change observed in one of the replicates of 0–2 cm layer, site Sh, is shown.

With equation 5, the constant of methane oxidation rate *k*′ was calculated from *k* which was obtained by approximating the change in methane concentration with an exponential function based on equation 4 (Fig. 1).

### DNA extraction

DNA was extracted from each sediment sample (0.84–1.07 g fresh weight) using the Ultra Clean Soil DNA kit (MoBio Laboratories, Carlsbad, CA, USA) according to the manufacturer’s instructions for maximum yields. The DNA solutions were stored at −30°C until quantative real-time PCR (q-PCR) and PCR-denaturing gradient gel electrophoresis (DGGE) analyses.

### Quantification of the pmoA gene

Copy numbers of the *pmoA* gene were determined by q-PCR with the primer pair A189/mb661r (4, 11) targeting the *pmoA* gene of both type I and II MOB. The conditions for q-PCR were the same as those described previously (35). To evaluate the effects of PCR inhibitors, the *pmoA* gene copy numbers in serially diluted DNA solutions of each sample were determined. Nearly identical estimates were obtained using the dilution series (data not shown), indicating that the effects of PCR inhibitors on quantification were not significant. The copy number per sediment volume was calculated from the copy number obtained by q-PCR using DNA solution diluted 100-fold from the original extract, and the volume density of each sediment section.

### PCR-DGGE

PCR-DGGE analyses were conducted for the *pmoA* and 16S rRNA genes. For amplification of the *pmoA* gene, primer set A189/mb661r was used with the addition of a GC-clamp at the 5′-end of the A189 primer (9). PCR amplification was initiated with denaturation for 5 min at 94°C. Each PCR cycle consisted of 30 s of denaturation at 94°C, 30 s of annealing at 56°C and 45 s of extension at 72°C. The total number of cycles was 34, and additional extension was carried out for 10 min at 72°C. For 16S rRNA genes of aerobic MOB, nested-PCR was carried out. In the first round of nested PCR, primer pairs MethT1dF/MethT1bR and 27F/MethT2R were used for specific amplification of type I and type II MOB, respectively (39). The PCR products obtained with the specific primer pairs were used as templates for the second PCR using the primer pair GC341F/907R (21). The conditions for nested PCR were the same as those described previously (17). The final PCR products were subjected to DGGE analysis, as described previously (17), with some modifications described below. The range of denaturant gradient was 35–60% (100% corresponds to 40% [v/v] formamide and 7 M urea), and electrophoresis was performed at 75 V for 16 h. The predominant DGGE bands were excised from the lanes representing the 0–2 cm sediment layer on both sites. The DGGE bands were also sequenced as described previously (17).

### Phylogenetic analysis

The *pmoA* sequence obtained from the DGGE band was translated to an amino acid sequence using MEGA4 (31). The amino acid sequence was aligned with related sequences obtained from the Genbank database using the ClustalX program (33). Genetic distances were calculated using the Poisson correction, and the phylogenetic tree was constructed with the minimum evolution method using MEGA4 software. The robustness of the resultant tree was examined with bootstrap tests of 1000 replicates. Sequences of the 16S rRNA gene were analyzed using the Ribosomal Database Project classifier (36) to deduce phylogenetic positions. The sequences of the 16S rRNA gene were also compared to those in the DDBJ/EMBL/GenBank database using the online BLAST program on the NCBI website.

### Nucleotide sequence accession numbers

The nucleotide sequences determined in this study have been deposited in the DDBJ/EMBL/GenBank and assigned accession numbers AB665482–AB665495.

## Results

### Vertical profiles of methane concentration, potential activity, and *pmoA* gene copy number

**Fig. 2:**
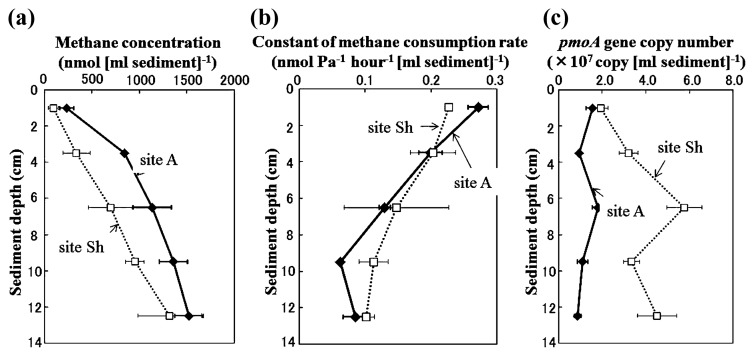
Vertical profiles of methane concentration (A), constant of aerobic methane oxidizing potential (B) and *pmoA* gene copy number (C) at sediments at site A (closed diamonds) and site Sh (open squares). Bars indicate standard deviation of triplicate samples, except for potential activities in 5–8 cm and 11–14 cm layers at site Sh measured in duplicate.

Methane concentration was higher at site A than site Sh in all sediment sections (Fig. 2A). On both sites, the methane concentration increased from the sediment surface to a depth of 11–14 cm. Potential activity of aerobic methane oxidation was observed at all sampling depths, but decreased linearly with increasing depth at both sites (Fig. 2B). The *pmoA* gene was also detected in all samples (from 8.8×10^6^ to 5.8×10^7^ copies/mL sediment, Fig. 2C). Peak copy number was observed at a depth of 5–8 cm at both sites and was higher at site Sh than site A at all sediment depths.

### DGGE analysis of MOB

**Fig. 3:**
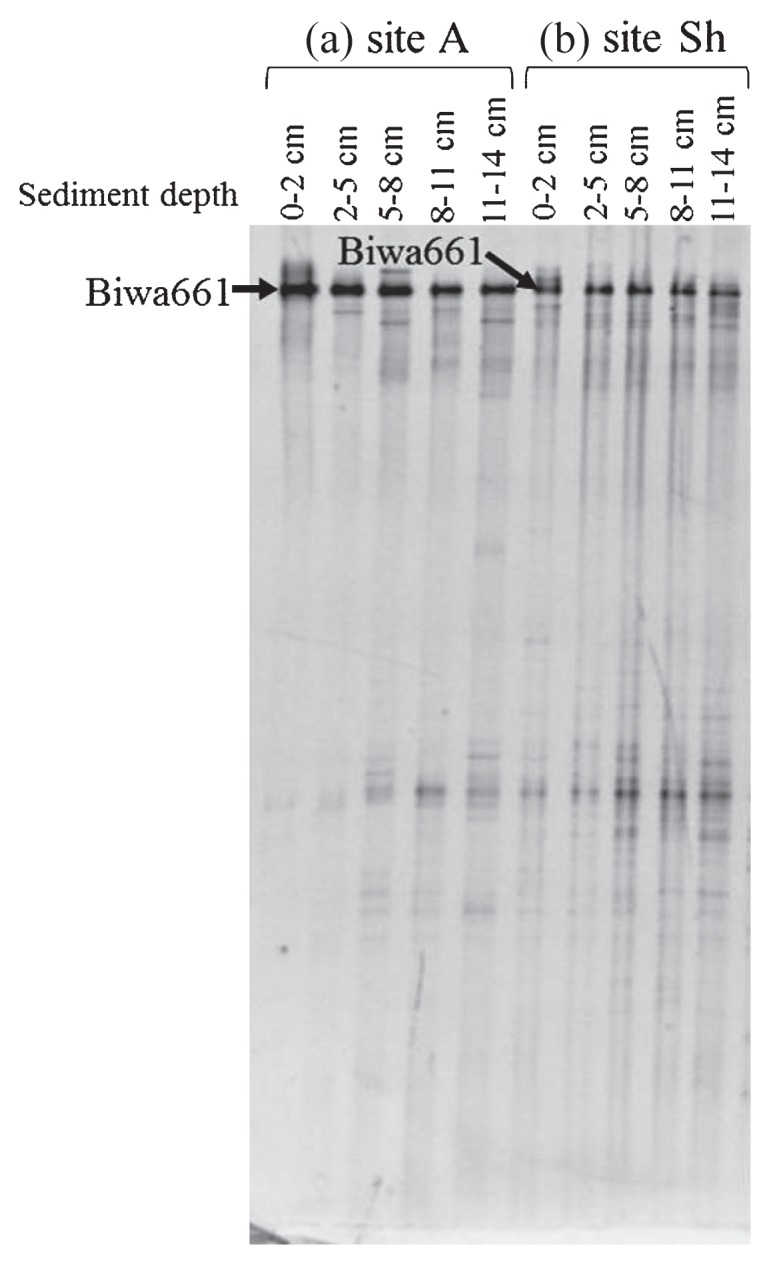
DGGE patterns of the *pmoA* gene. Bands indicated with an arrow were sequenced.

**Fig. 4:**
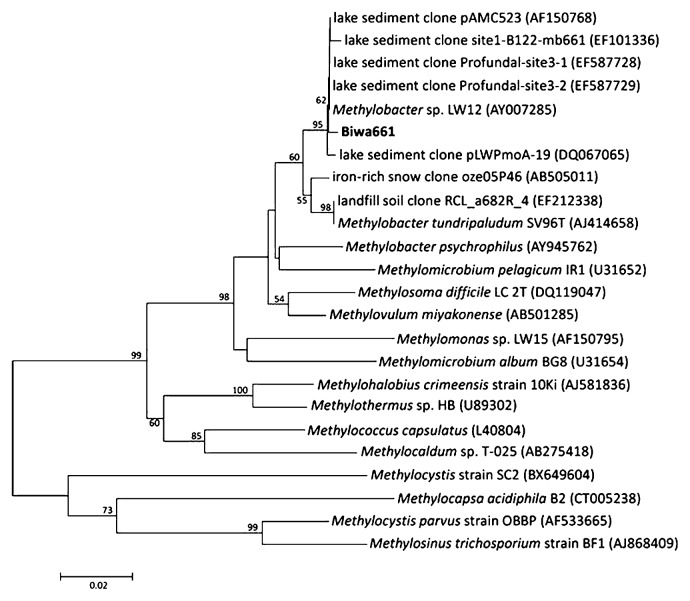
Tree showing the phylogenetic position of the amino acid sequence deduced from the sequence of the DGGE band (Fig. 3). This tree was constructed with 149 amino acid sites. Bootstrap values >50% (1000 replicates) are indicated at nodes.

The results of DGGE targeting the *pmoA* gene showed a simple banding pattern with a single predominant band, Biwa661, which was consistently observed in all the sediment layers at both sites (Fig. 3). The sequence of band Biwa661 was affiliated with that of the genus *Methylobacter* (Fig. 4) and was closely related to the sequences of clones obtained from freshwater lake sediments (4, 22, 26).

**Fig. 5:**
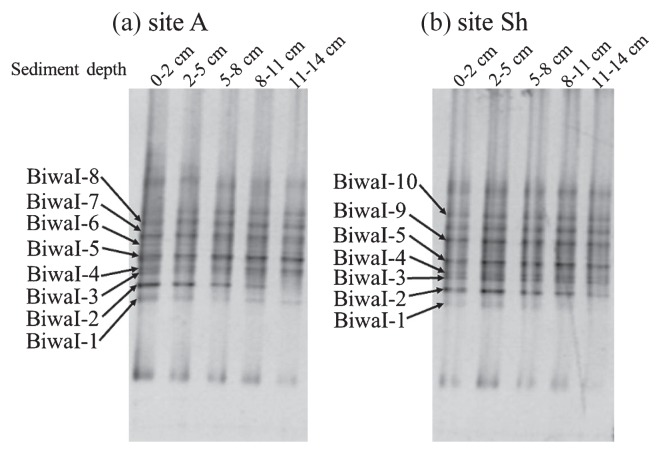
DGGE patterns of 16S rRNA gene of type I MOB. Bands indicated with arrow were sequenced.

Band patterns of DGGE targeting 16S rRNA genes of type I MOB were similar at the two sites (Fig. 5). The DGGE patterns did not change markedly with depth. The sequences of all predominant bands were closely related to those of the genus *Methylobacter* and clones obtained from permafrost soil, sediment, aquifer, lake water, iron rich snow, hydrothermal vent water, rice paddy and activated sludge (12, 16–18, 22, 23, 25, 26).

**Fig. 6:**
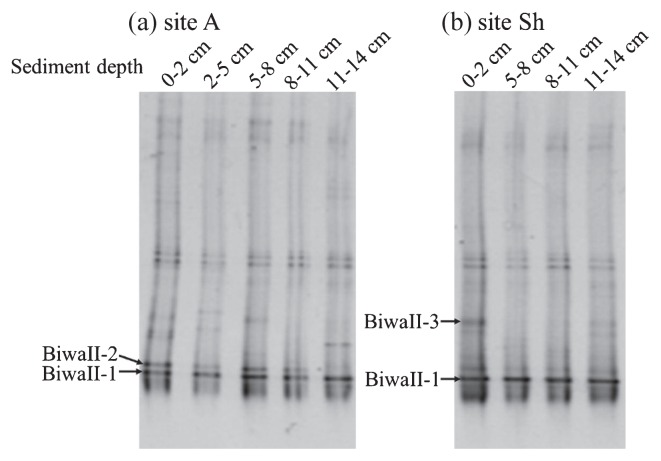
DGGE patterns of 16S rRNA gene of type II MOB. Bands indicated with arrow were sequenced.

Similar results of an almost unchanged band pattern were also obtained with the primer pair targeting type II MOB (Fig. 6). The obtained sequences of type II MOB were closely related to those of isolates of the genus *Methylocystis* and clones obtained from aquifers, methane-oxidizing reactors, reservoir water, drinking water, human skin, sediments and rice paddies (7, 23, 25, 37, 38).

## Discussion

In the present study, vertical profiles of the potential activity and copy number of the *pmoA* gene exhibited clearly different patterns at both sites. Judging from the results of DGGE, the discrepancy between gene abundance and potential activity cannot be attributed to depth-related changes in community structure. Under the experimental conditions applied in this study, an oxygen concentration higher than that in the sediment may have inhibited MOB activity (10, 28). Even if such an effect existed, it would have been imposed evenly on the samples in all layers because they shared major constituents of the MOB community. Therefore, the abundance of the *pmoA* gene may not reflect the number of MOB cells retaining potential activity in the anoxic sediment. In contrast to the results of DGGE in this study, depth-related changes in planktonic MOB community in response to shifts in environmental conditions have been clearly shown in studies performed in a stratified lake (17, 35). Furthermore, pronounced shifts in the MOB community in response to changes in oxygen and methane concentrations have been reported in a study of enrichment culture experiments (10). The similar DGGE band patterns exhibited by the sediment surface and deeper layers suggest that aerobic MOB that had grown on the oxic sediment surface had been buried in the deep anoxic sediment, and were thus detected in DNA-based analysis. This speculation may also explain the observed discrepancy between gene abundance and activity. Decreased activity with increasing sediment depth would reflect loss of activity during prolonged burial in sediment.

Assuming that the aerobic MOB detected in the anoxic sediment were not growing there, vertical profiles of MOB detected in DNA-based analysis can be interpreted as historical changes in MOB community. In a previous study, depth-related distribution of MOB in sediment was analyzed in an Antarctic lake, Ace Lake, which went through stages of being a freshwater lake, marine inlet, and saline meromictic lake (3). In the analysis of the 16S rRNA gene, no MOB were detected in the sediment layers corresponding to the marine period, in contrast to the preceding and following stages (3). Similar to the study of Ace Lake, the quantitative changes of the MOB gene observed in this study may be explained by environmental changes in Lake Biwa. It has been shown that environmental changes in this lake during the past several decades are reflected in phytoplankton buried in the sediment, from the surface to a depth of 15 cm (34).

The *pmoA* gene copy number was higher in the sediment from site Sh than from site A at all sediment depths (Fig. 2C). Although it is impossible to identify factors that contributed to this difference without appropriate replication, a difference in water depth may be one of the important factors. In Lake Biwa, internal waves occur at a water depth of 20 m to 45 m (20). Internal waves are known to lead to increases in methane, phosphorous, and nitrate in the overlying waters of the sediment (20, 29, 30). Such internal wave-enhanced substance transport may be favorable for surface-dwelling MOB that depend on methane produced in deeper sediments, resulting in a higher *pmoA* gene copy number in the region of corresponding water depth.

The results of the present study suggest that genes of MOB retrieved from anoxic sediments do not necessarily reflect active aerobic MOB cells, and the *pmoA* gene copy number may not serve as an indicator of active MOB abundance in deeper sediments. However, MOB activity in deeper sediments was not examined under *in situ* conditions in this study. Higher abundance of the *pmoA* gene in the anoxic sediment relative to the surface layer was also observed in Lake Constance (27). In that study, the highest *in situ* methane oxidation activity derived from high-resolution methane profiles was also observed at the depth where no oxygen was detected. Methane oxidation by members of the genus *Methylobacter* under conditions of undetectable oxygen was also suggested in another recent study performed in a meromictic lake (2). Further studies on the *in situ* activity of MOB in deep sediments will be needed to obtain an accurate picture of methane dynamics in freshwater lakes.
